# ARCH Is Bringing Asia Closer to the Rest of the World

**DOI:** 10.3390/brainsci13101430

**Published:** 2023-10-08

**Authors:** Tissa Wijeratne, Krishnamurthy Ravishankar

**Affiliations:** 1 Asian Regional Consortium for Headache (ARCH), Australia Institute of Migraine, 522, Bell Street, Pascoe Vale South, VIC 3044, Australia; 2 Asian Regional Consortium for Headache (ARCH), Jaslok and Lilavati Hospitals, Mumbai 400050, India; dr.k.ravishankar@gmail.com

As the immediate Past President (KR) of Asian Regional Consortium For Headache (ARCH) and the newly appointed President (TW) of ARCH, we are pleased to share our thoughts with the readers of MDPI.

Together, as Chair and Co-Chair, we have just completed the ninth ARCH meeting with a new record attendance for Asia. Due to unforeseen reasons, we had only four weeks of lead time before the virtual congress that was held on 28 and 29 July 2022. Over these four weeks, we exchanged well over a thousand email messages, WhatsApp texts and zoom calls between India and Australia, where we were based. The result of all this quick co-ordination was a high-quality collection of over fifty recorded lectures from experts. The Faculty included seven past, current and future Presidents of The IHS and four past, present and future ARCH Presidents, thus representing global leaders in headache medicine. TW handled the entire technical aspect which included the collection of recordings, the conference website, the webcasts and delivery worldwide. KR provided daily guidance to the Organising Team from Australia Institute of Migraine (www.instituteofmigraine.com.au accessed on 15 September 2023) and Migraine Foundation Australia (www.migrainefoundation.org.au accessed on 15 September 2023) with ample support, given his vast experience and wisdom. We initially expected to host only 100–150 headache experts and trainees from Asia. The conference registration site was launched on 22 July with the plan to unleash the opening ceremony on 28 July at 15.00 AEST with free entry. The International Headache Society (IHS) first mailshot was sent out on 21 July.

To our surprise and satisfaction, approximately 1400 delegates registered by 28th July with a new record in the entire history of ARCH. The event commenced on Day 1 with a comprehensive presentation by Professor David Dodick on pharmacological advances in headache medicine from 2017–2022. This was followed by a similar talk on future pharmacological advances in headache medicine from 2022–2027 by Professor Peter Goadsby. KR and TW chaired the session on Brain Decade with a live Question and Answer session that was moderated by Professor Min Kyung Chu. 

The virtual conference was delivered over two days covering all other current aspects of headache medicine. The feedback received from the delegates was excellent.

**It is worth reflecting on the key lessons from the history-making Ninth ARCH2022 Meeting as we continue to move towards the rest of the world**

Through the success of this meeting, KR and TW showed that the power of collaboration, cooperation and communication is a magic recipe to improve access and awareness in Headache Education. Well over fifty high-quality lectures were delivered to the delegates at no cost due to the IHS support. One thousand four hundred clinicians were gifted with high-quality educational material through www.archhub.org accessed on 15 September 2023.

We witnessed the joy of ARCH members through their active online participation during the two-day congress. ARCH is an ideal platform and stage for us to interact with each other for training, learning, and discovery. ARCH will also work towards an open-access headache journal for the region.

ARCH—Past, Present and Future.

Asia–Oceania is home to 4.7 billion people. Nearly 64% of the global population lives here. ARCH has an enormous responsibility for the billions of headache sufferers, clinicians and researchers in the region. The history of ARCH dates back to 2004. K. Ravishankar (India), Charles Siow (Singapore), Shuu-Jiun Wang (Taiwan), Chin-San Chung (Korea), Raymond Tak Fai Cheung (Hong Kong), Siwaporn Chankrachang (Thailand), Julia Shahnaz Merican (Malaysia) and Fumihiko Sakai (Japan) met in Kuala Lumpur for the Migraine Assessment for Physicians Survey meeting. This team was excited about the prospect of IHC2005 coming for the first time to Kyoto and saw this as an unprecedented opportunity to set things right in Asia. The need for a special regional organisation to promote headache care and awareness was discussed in detail. This culminated in the birth of the Asian Headache Foundation (AHF) in 2005. Oceania was not part of AHF for a few more years. AHF underwent several name changes as the group gathered on a biannual basis. The organisation was renamed as ARCH (Asian Regional Consortium for Headache) in 2018. 

ARCH 2022 has now added four other nations in Asia–Oceania to the membership and has now has 19 member countries—China, India, Japan, Thailand, Taiwan, Malaysia, Laos, Myanmar, Nepal, The Philippines, South Korea, Singapore, Australia, New Zealand, Bangladesh, Pakistan, Indonesia, and Sri Lanka. Asia–Oceania is home to 53 countries. We must work hard to recruit more countries and expand ARCH.

**Collaboration, Communication and Co—Operation to Improve Access**

Brain disorders are a leading cause of disability. The cost of brain conditions such as anxiety, depression and headache disorders to the world is more than that of the entire cancer and cardiovascular disorders according to the WHO health statistics. Headache disorders are responsible for 17% of the disability out of all brain disorders. Raising awareness and advocating for better headache care in Asia is an urgent need.

The young generation is our future. TW, the new President of ARCH, hopes to inspire medical students and neurology trainees through a hybrid Asia–HEAD program targeting needy regions in Asia–Oceania in the coming years. In due course, he plans to also unleash a regular virtual session on the Inspiring People in Headache medicine series in collaboration with The IHS and the Australian Institute of Migraine.

The recent adoption of WHO-IGAP calls for ongoing collaboration, communication and cooperation between organisations to promote quality neurology and better brain health. The immense benefit of IHS-ARCH cooperation, collaboration and communication were critically important for the success of this historic ninth ARCH2022—Bringing Asia Closer to the rest of the world. We will work with ample synergy with IHS, WFN and WHO to promote quality neurology and better brain health for our patients with headache disorders in Asia and Oceania (refer to the Supplementary Material Figure S1 and Video S1. 

## Supplementary Materials

The following are available online at https://www.mdpi.com/article/10.3390/brainsci13101430/s1, Figure S1: ARCH National leaders from Asia at the IHC2023 Seoul, Video S1: ARCH is bringing Asia closer to the rest of the world.

