# Supplementary figures and images for: ARCH Is Bringing Asia Closer to the Rest of the World
Source: Brain Sci. 2023 Oct 8;13(10):1430. doi: 10.3390/brainsci13101430 (PMC10605090; doi:10.3390/brainsci13101430)

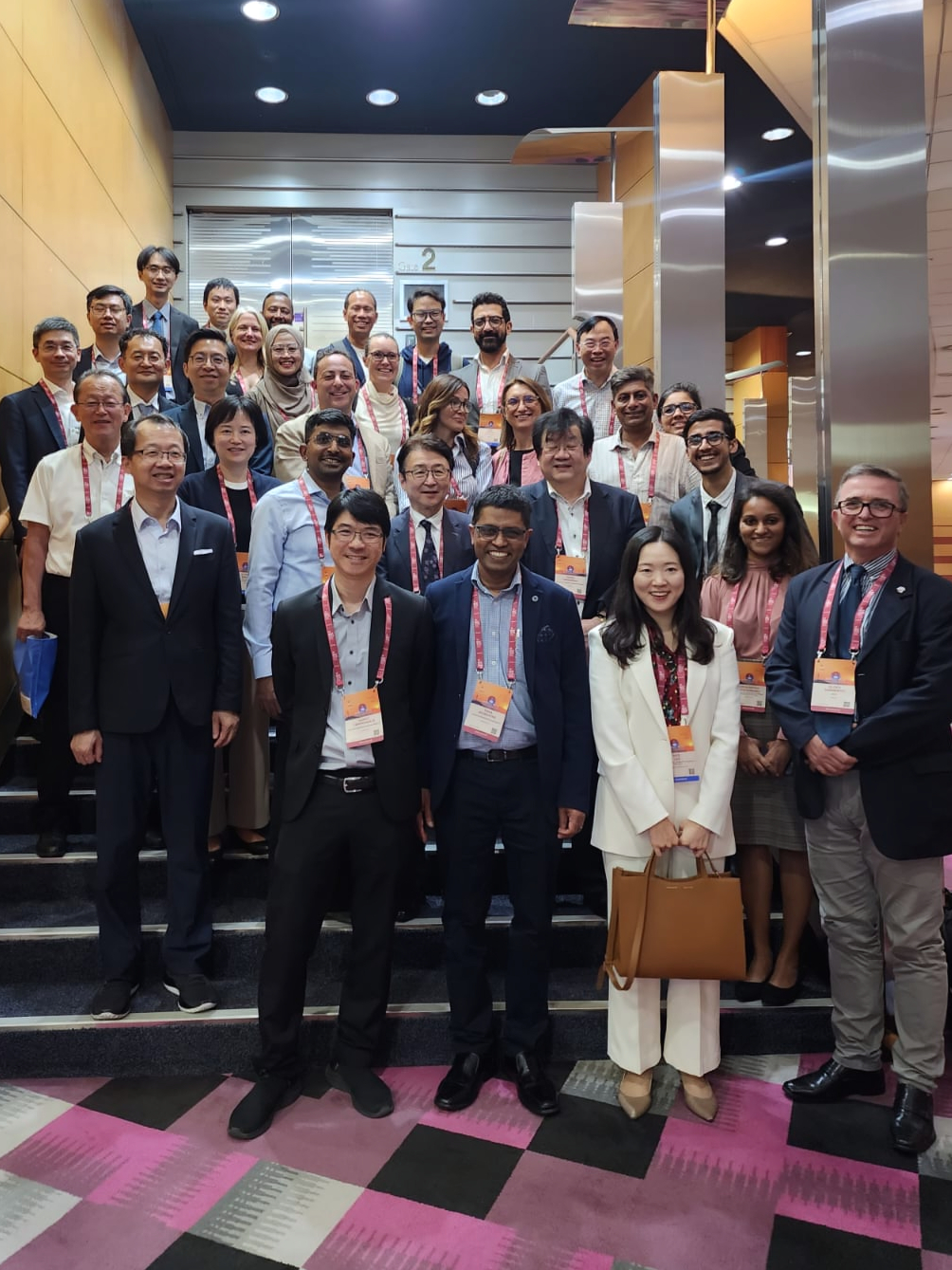

Supplement: Supplementary file 1 [file brainsci-13-01430-s001.zip › supplementary material/ARCH National leaders from Asia at the IHC2023 Seoul.jpg]
